# Hormone concentrations throughout uncomplicated pregnancies: a longitudinal study

**DOI:** 10.1186/s12884-016-0937-5

**Published:** 2016-07-04

**Authors:** Helena Schock, Anne Zeleniuch-Jacquotte, Eva Lundin, Kjell Grankvist, Hans-Åke Lakso, Annika Idahl, Matti Lehtinen, Heljä-Marja Surcel, Renée T. Fortner

**Affiliations:** Division of Cancer Epidemiology, German Cancer Research Center, Im Neuenheimer Feld 280, Heidelberg, 69120 Germany; Department of Medical Biosciences, Umeå University, Umeå, Sweden; Department of Population Health, New York University School of Medicine, New York, USA; New York University Cancer Institute, New York University School of Medicine, New York, USA; Department of Clinical Sciences, Obstetrics and Gynecology, Umeå University, Umeå, Sweden; School of Public Health, University of Tampere, Tampere, Finland; Unit of Sexual and Reproductive Health, National Institute for Health and Welfare, Oulu, Finland

**Keywords:** Longitudinal study, Pregnancy, Steroid hormones, OPG, sRANKL

## Abstract

**Background:**

Evidence suggests that the hormonal milieu of pregnancy is an important determinant of subsequent cancer and other chronic diseases in both the mother and the offspring. Many of the existing maternity and birth cohorts include specimens drawn only once during pregnancy. How well a single blood specimen collected during a pregnancy characterizes exposure to these hormones throughout gestation, and also in subsequent pregnancies, is not well understood.

**Methods:**

We used serial serum samples from 71 pregnant women (25 primiparous, 25 multiparous, and 21 with two consecutive pregnancies) with natural, complication-free pregnancies and a healthy offspring at term who participated in a population-based screening trial for congenital infections in Finland between January 1st, 1988 and June 30, 1989 and provided a blood sample in each trimester.

**Results:**

Hormone levels were more strongly correlated between consecutive trimesters of a pregnancy than between the 1st and 3rd trimester (e.g., estradiol, r_T1 vs. T2_ = 0.51 and r_T2 vs. T3_ = 0.60, *p* < 0.01; r_T1 vs. T3_ = 0.32, *p* < 0.05). Concentrations of sRANKL remained stable throughout gestation, whereas estradiol, estrone, progesterone, testosterone, prolactin, and osteoprotegerin increased throughout pregnancy. First trimester hormone concentrations explained less of the variation in the third trimester on their own than second trimester hormone levels (e.g. estradiol R^2^_T1_ *=* 16 % and R^2^_T2_ = 42 %). Addition of maternal (e.g., smoking) and/or child characteristics (e.g., sex) improved the accuracy of the 3rd trimester estimates for some of the hormones.

**Conclusions:**

One hormone measurement in early pregnancy, in conjunction with maternal and fetal characteristics, permits estimation of 3rd trimester hormone concentrations. Therefore, single hormone measurements available from maternity cohorts are suitable to quantify hormone exposure during pregnancy. To our knowledge, we provide the first data on correlations between hormone concentrations both across trimesters of a single pregnancy, as well as between two subsequent pregnancies.

**Electronic supplementary material:**

The online version of this article (doi:10.1186/s12884-016-0937-5) contains supplementary material, which is available to authorized users.

## Background

The hormonal milieu of pregnancy may be an important determinant of subsequent cancer and other chronic diseases both in the mother and the offspring [1, 2].

Depending on the outcome of interest, hormone concentrations measured at different periods during pregnancy may be of relevance. For instance, hormones measured in early pregnancy, during which fetal organogenesis takes place, may be important when neurodevelopmental conditions in the offspring are of interest, whereas hormone concentrations during late pregnancy may be relevant in relation to maternal risk of breast and ovarian cancers, given evidence that complete pregnancies are associated with these malignancies [3, 4].

Established biorepositories in Northern Europe (e.g., Finland) store sera collected during early pregnancy (predominantly in weeks 8 to 14) for screening of systemic infections such as HIV, hepatitis, and/or rubella. Worldwide, maternity and birth cohorts have been established to study associations between pregnancy and early-life exposures with health and disease outcomes in the mother and/or the offspring. Many of the existing cohorts include specimens drawn once during the first half of pregnancy [5–9], with few collections obtaining a blood sample later in pregnancy [10, 11]. Furthermore, only few studies obtained blood in consecutive trimesters from sizeable cohorts of pregnant women [12–14]. For many outcomes (e.g., chronic diseases in the off-spring in adulthood), a large study population is necessary as cohort members must be followed as long as decades to accumulate a sufficient number of cases for an investigation.

Whether a hormonal measurement from a single blood specimen collected early during a complication-free pregnancy is representative of exposure throughout gestation, or exposure in a subsequent pregnancy, is not established. From a research perspective, this knowledge could help optimize the use of existing maternity cohorts in which only specimens drawn once during the first half of pregnancy are available.

Therefore, the aim of our study was to explore the correlations between hormone concentrations measured in serum during the 1st, 2nd, and 3rd trimester of (1) a single uncomplicated pregnancy, (2) two subsequent uncomplicated pregnancies, and (3) to calculate how well 1st and/or 2nd trimester hormone levels estimate concentrations during 3rd trimester in an uncomplicated pregnancy. We measured sex steroids (estradiol, estrone, progesterone, and testosterone) and prolactin that are implicated in the development of maternal breast and ovarian cancer [1] and two new biomarkers (osteoprotegerin [OPG] and soluble receptor activator of nuclear factor kappaB ligand [sRANKL]) that have not yet been studied in detail.

## Methods

We used serum samples from pregnant women collected in a population-based screening trial for congenital infections between January 1st, 1988 and June 30, 1989 in the 76 maternity centers in the metropolitan Helsinki (Finland) area. All pregnant women aged 15–45 years (*n* = 18,616) were invited to donate a blood sample during the 1st, 2nd, and 3rd trimesters of a pregnancy [14].

Among the 16,793 participants (90 %) who donated three serum samples we first selected women with a singleton, healthy offspring at term resulting from a natural, uncomplicated pregnancy. Women with an uncomplicated pregnancy were defined as women not being diagnosed with pre-eclampsia or hypertension during pregnancy and not being hospitalized due to threatening premature delivery or bleeding. In a second step, we randomly selected 25 women giving birth to their first child (primipara) and 25 women giving birth to their second child (multipara), aged 20–34 years and whose blood samples were obtained at weeks 10–12 (T_1_), weeks 20–22 (T_2_), and weeks 35–37 (T_3_) of one pregnancy. Additionally, 21 women with two consecutive singleton pregnancies during the study period were included. These women donated samples for both pregnancies in gestational weeks 8–14, 18–24, and 33–38. All blood samples were processed using a standardized protocol and stored at −20 °C [14].

Characteristics related to pregnancy (e.g., smoking during early pregnancy, pregnancy length, parity) and to the newborn (e.g., gender, birth weight and birth length) were obtained through linkages from the Finnish birth registry.

The study was approved by the ethical committees of the National Institute for Health and Welfare, Finland, University of Umeå, Sweden, and University of Heidelberg, Germany.

### Laboratory analyses

All hormonal analyses were performed at the Clinical Chemistry Laboratory of Umeå University Hospital, Umeå, Sweden. Serum specimens from each woman were assayed in the same laboratory run. In addition to routine laboratory quality controls, a pool of serum was created at the beginning of the study and two aliquots, undistinguishable from the study samples were inserted in each laboratory run. All reported intra- and inter-assay coefficients of variation (CV) are based on the blinded pool quality controls.

Serum concentrations of sex steroids were quantified by high-performance liquid chromatography tandem mass spectrometry (HPLC-MS) on an Applied Biosystems API4000 triple stage quadrupole mass spectrometer. Serum prolactin (PRL) concentrations were measured by immunoassay (Elecsys Prolactin II, Roche, Mannheim, Germany). The determination of serum osteoprotegerin (OPG) concentrations was performed by enzyme-linked immunosorbent assay (TNFRSF11B (Human) ELISA Kit, Abnova). Serum soluble RANK ligand (sRANKL) concentrations were determined by immunoassay (ampli-sRANKL, Biomedica, Vienna, Austria).

Intra- and inter-assay CVs were <18 % for all hormones with the exception of testosterone. The intra-assay CV for testosterone was 12 %, whereas the inter-assay CV was 44 %. After removing a single batch for testosterone, the inter-assay CV decreased to 12 %.

Out of 150 samples, sRANKL was below the detection limit in 21 samples, OPG was below the detection limit in one sample, and prolactin was below the detection limit in four samples.

Sex steroid hormones were measured in all samples, whereas PRL, OPG, and sRANKL were measured only in samples from women who gave birth once during the study period.

### Statistical analyses

Women with one pregnancy were analyzed separately from those with two pregnancies.

Spearman's rank correlation coefficient was used to assess the correlations between the investigated hormones at the same point in time as well as between-trimester correlations for each hormone, and to measure the correlation of hormone concentrations between two consecutive pregnancies in the same woman. To test for differences between the correlation coefficients in the primiparous and multiparous women each correlation coefficient was converted into a z-score using Fisher's r-to-z transformation. The z-scores were then compared taking into account the sample sizes employed to obtain each coefficient [15]. We considered adjustment for multiple comparison using the Bonferroni method (p_Bonf_ < 0.05/145 = 0.0003) as a secondary analysis.

We calculated median percentage changes in maternal hormone concentrations from the 1st to 3rd trimester and used the Kruskal Wallis test (p_KW_) to test the differences in hormone concentrations between the trimesters. The Mann–Whitney-*U* test (p_MW_) was used to assess whether hormone concentrations in the three trimesters were significantly different by parity (primipara vs. multipara), smoking status at first blood draw (current vs. no), and fetal sex (male vs. female).

The percentage changes in the 3rd trimester hormone concentrations were estimated per 1 % increase in hormone levels measured in the 1st and/or 2nd trimester, adjusted for gestational age. Maternal and newborn characteristics were added to these models one at a time to assess their individual contribution to 3rd trimester hormone concentrations.

We used the Wilcoxon signed rank sum test (p_WS_) to compare differences in hormone concentrations between trimesters of the two consecutive pregnancies.

For the study population of women with one full-term pregnancy, we conducted sensitivity analyses limited to women who were pregnant for the first (primigravid; *n* = 19) or second time (multigravid; *n* = 19) to assess whether observed differences between primiparous and multiparous women were due to gravidity or parity. Analyses on sRANKL were repeated replacing the missing values with the lower limit of detection of the assay (0.02 pmol/L).

All statistical tests were two-sided and considered significant at *p* < 0.05. Statistical analyses were performed with the Statistical Analyses System (SAS), version 9.3 (SAS Institute, Inc., Cary, North Carolina).

## Results

The baseline characteristics of all study participants are presented in Table 1. Maternal and child characteristics were similar between primiparous women, multiparous women, and women participating with two consecutive pregnancies. Overall, the majority of study participants did not smoke during pregnancy (*n* = 52; 73 %) and more pregnancies ended in the delivery of a boy (*n* = 51; 55 %) than a girl (*n* = 41; 45 %).

**Table 1:** Baseline characteristics; median (range), or n (%)

	One pregnancy during study period		Two consecutive pregnancies during study period	
Characteristics	Primiparous (*n* = 25)	Multiparous (*n* = 25)	Pregnancy 1 (*n* = 21)	Pregnancy 2 (*n* = 21)
Maternal age, years	27.2 (20.6–33.5)	27.5 (20.5–33.1)	26.5 (17.8–42.7)	27.7 (18.6–44.0)
Pregnancy length, days	282 (255–296)	283 (266–292)	279 (258–294)	278 (262–291)
Parity				
0	25 (100 %)		13 (62 %)	
1		25 (100 %)	6 (29 %)	13 (62 %)
2			2 (9 %)	6 (29 %)
3				2 (9 %)
Child weight, kg	3.3 (2.4–4.7)	3.8 (2.8–4.6)	3.5 (2.8–4.5)	3.8 (2.9–4.3)
Child length, cm	49 (45–55)	51 (47–54)	50 (47–53)	50 (49–53)
Child sex				
Boy	10 (40 %)	13 (52 %)	14 (67 %)	14 (67 %)
Girl	15 (60 %)	12 (48 %)	7 (33 %)	7 (33 %)
Maternal smoking				
No	19 (76 %)	20 (91 %)	13 (62 %)	13 (65 %)
Yes	6 (24 %)	2 (9 %)	8 (38 %)	7 (35 %)

### Associations across trimesters of one pregnancy

Hormones of placental origin (estradiol, estrone, and progesterone) were highly correlated with each other (*r* > 0.72, *p* < 0.0001) and with PRL (*r* > 0.68, *p* < 0.0001; data not shown). More modest correlations were observed for OPG with PRL (*r* = 0.51), estrone (*r* = 0.52), estradiol (*r* = 0.59), and progesterone (*r* = 0.61; p for all <0.0001). Testosterone and progesterone were positively correlated (*r* = 0.34, *p* < 0.0001), whereas testosterone and sRANKL were negatively correlated (*r* = −0.30, *p* = 0.001). Gestational age was highly correlated with OPG, PRL, estrone, progesterone, and estradiol (*r* = 0.63 (OPG) to *r* = 0.89 (estradiol); *p* < 0.0001). Weak, borderline significant correlations in the 2nd and 3rd trimesters were observed for OPG with child length (r_T2_ = 0.33, r_T3_ = 0.28; *p* ≤ 0.05) and child weight (r_T2_ = 0.24, r_T3_ = 0.26; p_T3_ ≤ 0.10). Irrespective of trimester, sRANKL was not significantly correlated with child length (e.g., r_T3_ = 0.20, p_T3_ = 0.20) or child weight (e.g., r_T3_ = 0.11, p_T3_ = 0.47; data not shown).

Hormone concentrations were more strongly correlated between consecutive trimesters of a pregnancy than between the 1st and 3rd trimester (e.g. estradiol, r_T1 vs. T2_ = 0.51 and r_T2 vs. T3_ = 0.60, *p* < 0.01; r_T1 vs. T3_ = 0.32, *p* < 0.05; Table 2). Correlation coefficients in the primiparous and multiparous women were significantly different for estrone (p_T1 vs. T3_ = 0.01) and sRANKL (p_T1 vs. T2_ < 0.001; Table 2). Using the Bonferroni corrected p-value of 0.0003 (p_Bonf_ = 0.05/145), correlation coefficients remained significant between hormones and with GA, as well as between the trimesters of a single pregnancy overall and for sRANKL in primiparous and multiparous women.

**Table 2:** Spearman correlation coefficients adjusted for gestational age between successive trimesters of one pregnancy ^a^

	Overall		Primiparous		Multiparous	
Trimester	1st	2nd	1st	2nd	1st	2nd
Estradiol						
2nd	0.509 ^**^		0.518 ^***^		0.519 ^***^	
3rd	0.319 ^***^	0.597 ^*^	0.376	0.629 ^**^	0.121	0.519 ^***^
Estrone						
2nd	0.519 ^**^		**0.742** ^*^		**0.307**	
3rd	0.293	0.609 ^*^	**0.592** ^**^	0.707 ^**^	**−0.107**	0.521 ^***^
Progesterone						
2nd	0.629 ^*^		0.306		0.719 ^*^	
3rd	0.394 ^**^	0.643 ^*^	0.321	0.569 ^***^	0.323	0.672 ^**^
Testosterone						
2nd	0.674 ^*^		0.789 ^*^		0.511 ^***^	
3rd	0.508 ^**^	0.765 ^*^	0.614 ^**^	0.850 ^*^	0.240	0.551 ^**^
Prolactin						
2nd	0.575 ^*^		0.739 ^*^		0.464 ^***^	
3rd	0.327 ^***^	0.686 ^*^	0.675 ^**^	0.660 ^**^	0.315	0.661 ^**^
OPG						
2nd	0.538 ^*^		0.631 ^**^		0.406	
3rd	0.497 ^**^	0.779 ^*^	0.574 ^***^	0.643 ^**^	0.441 ^***^	0.827 ^*^
sRANKL						
2nd	0.774 ^*^		**0.968** ^*^		**0.427**	
3rd	0.729 ^*^	0.818 ^*^	0.806 ^*^	0.878 ^*^	0.528 ^***^	0.808 ^*^

Significant *p*-values are indicated as ^*^ < 0.0003 (Bonferroni corrected *p*-value), ^**^ < 0.01, ^***^ < 0.05

^a^ Correlation coefficients in bold indicate a significant difference (*p* < 0.05) comparing primiparous vs. multiparous women

We examined median hormone concentrations across trimesters and the median %-change from 1st to 3rd trimester overall and by parity (Table 3). The 1st to the 3rd trimester median levels increased significantly (p_KW_ < 0.0001) 1.5-fold for OPG, 4-fold for progesterone, 6-fold for PRL, 9-fold for estradiol, and 10-fold for estrone. Testosterone and sRANKL concentrations did not change significantly across pregnancy.

**Table 3:** Median hormone concentrations (10–90th percentiles) overall and by parity ^a^

	All women (*n* = 50)	Primiparous (*n* = 25)	Multiparous (*n* = 25)	p_MW_^c^
Estradiol (ng/mL)				
1st trimester	2.18 (1.16–3.59)	2.11 (1.19–4.00)	2.42 (1.12–3.12)	0.92
2nd trimester	9.71 (5.33–15.1)	10.3 (5.20–14.8)	9.45 (5.46–15.3)	0.64
3rd trimester	20.4 (12.8–32.9)	22.3 (13.6–35.6)	19.5 (12.8–31.5)	0.71
Median %-change ^b^	876 %	858 %	889 %	
Estrone (ng/mL)				
1st trimester	0.93 (0.41–1.72)	0.71 (0.35–2.07)	1.11 (0.48–1.60)	0.14
2nd trimester	4.28 (1.99–8.54)	3.96 (1.64–8.84)	4.56 (2.77–6.88)	0.85
3rd trimester	11.5 (3.70–18.9)	10.4 (3.65–21.3)	11.7 (3.77–17.8)	0.92
Median %-change ^b^	976 %	919 %	1053 %	
Progesterone (ng/mL)				
1st trimester	25.6 (16.6–40.7)	27.3 (20.0–42.2)	23.0 (13.5–37.8)	0.03
2nd trimester	48.1 (31.6–78.5)	53.8 (37.8–85.2)	42.1 (28.7–71.3)	0.03
3rd trimester	130 (72.6–200)	138 (105–215)	107 (60.2–163)	0.007
Median %-change ^b^	411 %	422 %	396 %	
Testosterone (ng/mL)				
1st trimester	0.96 (0.42–1.98)	1.34 (0.78–2.15)	0.66 (0.36–1.11)	<0.0001
2nd trimester	1.19 (0.55–3.35)	1.98 (1.05–3.96)	0.73 (0.44–1.43)	<0.0001
3rd trimester	1.32 (0.50–4.07)	2.56 (0.54–6.06)	0.71 (0.50–2.41)	0.0008
Median %-change ^b^	61 %	74 %	38 %	
Prolactin (ng/mL)				
1st trimester	28.8 (16.3–57.6)	32.6 (19.8–63.3)	27.6 (10.6–47.0)	0.10
2nd trimester	126 (54.9–206)	105 (49.0–181)	139 (72–206)	0.32
3rd trimester	216 (124–318)	177 (115–258)	225 (133–320)	0.12
Median %-change ^b^	570 %	449 %	795 %	
OPG (pmol/L)				
1st trimester	3.79 (2.63–5.90)	3.45 (2.71–5.61)	3.93 (2.37–5.99)	0.21
2nd trimester	5.26 (3.10–7.91)	4.96 (3.28–7.15)	6.01 (3.10–9.01)	0.17
3rd trimester	9.74 (4.44–17.5)	8.18 (4.09–20.3)	10.5 (5.59–16.4)	0.12
Median %-change ^b^	143 %	112 %	162 %	
sRANKL (pmol/L)				
1st trimester	0.43 (0.10–0.81)	0.32 (0.06–0.92)	0.56 (0.31–0.74)	0.04
2nd trimester	0.43 (0.10–0.91)	0.31 (0.06–1.05)	0.51 (0.27–0.81)	0.26
3rd trimester	0.43 (0.20–0.82)	0.41 (0.14–0.95)	0.43 (0.30–0.72)	0.35
Median %-change ^b^	8 %	30 %	−14 %	

^a^ Conversion from ng/ml to nmol/l (SI units): estradiol*3.671, estrone*3.699, progesterone*3.18, testosterone*3.467, and prolactin*0.04348

^b^ Median %-change between 1st and 3rd trimester

^c^ *p*-value obtained from the Mann–Whitney-*U* test comparing primi- and multiparous women

**Fig 1:**
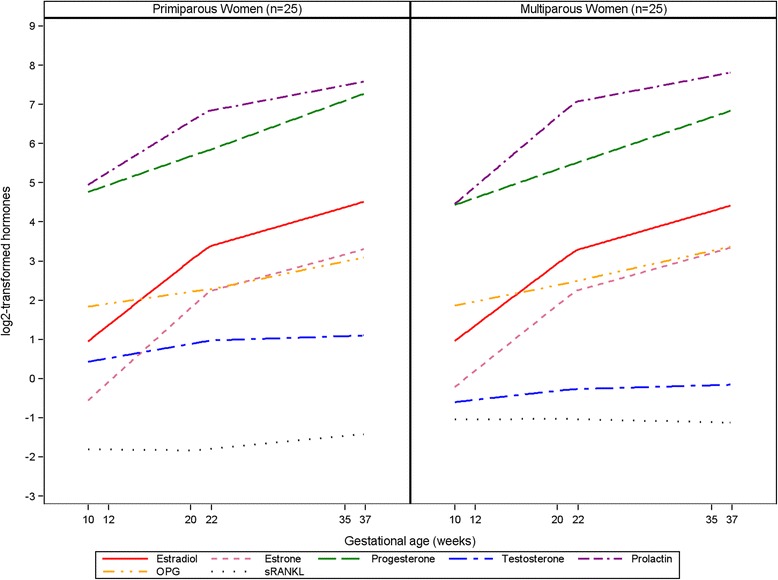
Trajectories of hormone concentrations in 25 primiparous and 25 multiparous women. Hormones are log_2_-transformed to fit in one graph

Progesterone and testosterone concentrations were lower in multiparous women as compared to primiparous women throughout gestation (e.g., progesterone T_3 primi_ = 138 ng/mL, T_3 multi_ = 107 ng/mL; p_MW_ = 0.007; testosterone T_3 primi_ = 2.56 ng/mL, T_3 multi_ = 0.71 ng/mL; p_MW_ = 0.0008). In contrast, concentrations of sRANKL were slightly higher in multiparous as compared to primiparous women, although this difference only reached statistical significance in the first trimester (T_1 primi_ = 0.32 pmol/L, T_1 multi_ = 0.56 pmol/L; p_MW_ = 0.04). Hormone concentration trajectories for primiparous and multiparous women are shown in Fig. 1.

Testosterone concentrations were higher throughout gestation in women who reported smoking in early pregnancy relative to non-smoking women (e.g. T_1 smoker_ = 1.69 ng/mL, T_1 non-smoker_ = 0.89 ng/mL; p_MW_ = 0.03). Third trimester concentrations of estradiol were higher in women reporting smoking in early pregnancy as compared to non-smoking women (T_3 smoker_ = 25.7 ng/mL, T_3 non-smoker_ = 18.6 ng/mL; p_MW_ = 0.01), whereas levels of PRL were lower (T_3 smoker_ = 136 ng/mL, T_3 non-smoker_ = 221 ng/mL; p_MW_ = 0.05). Lower PRL was observed in women carrying a boy as compared to women pregnant with a girl. However, this difference only reached statistical significance in the first trimester (T_1__boy_ = 23.7 ng/mL; T_1 girl_ = 32.7 ng/mL; p_MW_ = 0.02; T_2 boy_ = 101.6 ng/mL; T_2 girl_ = 147.4 ng/mL; p_MW_ = 0.09; T_3 boy_ = 175.3 ng/mL; T_3 girl_ = 222.4 ng/mL; p_MW_ = 0.38; data not shown).

We further examined the effect of a 1 % unit increase in 1st and/or 2nd trimester hormone concentrations on the estimates of 3rd trimester hormone concentrations.

Hormone concentrations measured in the 1st trimester accounted for 10 % (PRL) to 58 % (sRANKL) of the variation in the 3rd trimester (basic model, Table 4A). Women who were pregnant with their second child had significantly lower progesterone (24 %) but higher PRL (32 %) concentrations than primiparous women. Women who smoked at study enrollment had higher concentrations of estradiol (42 %) and testosterone (85 %), but lower PRL concentrations (41 %) compared to non-smoking women. Models that included all covariates and the 1st trimester hormone measurement (full-model, Table 4A) accounted for 7 % (progesterone) to 30 % (estradiol) more of the variation than a model based solely on the 1st trimester hormone level, with the exception of OPG (0 %) and sRANKL (-2 %).

**Table 4:** Percentage changes [95 % confidence intervals] in 3rd trimester hormone concentrations given 1st and 2nd trimester concentrations and maternal and newborn characteristics ^a^

	Estradiol	Estrone	Progesterone	Testosterone	Prolactin	OPG	sRANKL
A)							
1st trimester hormone only	26 [7, 50]	31 [7, 62]	34 [10, 63]	71 [36, 115]	16 [3, 31]	33 [13, 57]	52 [35, 71]
Adjusted R^2^ - basic model	16 %	10 %	13 %	31 %	10 %	18 %	58 %
1st trimester hormone							
Age at blood draw	−2 [−5, 1]	−1 [−7, 4]	0 [−3, 3]	−2 [−8, 5]	1 [−2, 4]	−2 [−5, 2]	−3 [−7, 1]
Smoking	42 [11, 82]	33 [−20, 113]	14 [−13, 45]	85 [2, 236]	−41 [−82, −9]	−12 [−61, 29]	−27 [−103, 25]
Parity	−5 [−28, 16]	−4 [−49, 38]	−24 [−50, −3]	−57 [−158, 4]	32 [9, 60]	21 [−6, 55]	−18 [−58, 14]
Child sex	27 [4, 54]	11 [−30, 59]	16 [−4, 40]	11 [−39, 71]	1 [−23, 27]	21 [−7, 56]	0 [−34, 34]
Birth length [per 5 cm]	−13 [−40, 9]	−39 [−102, 5]	−5 [−29, 17]	12 [−41, 76]	21 [−2, 51]	32 [0, 72]	12 [−23, 56]
Birth weight [per 100 g]	−1 [−2, 1]	−1 [−5, 2]	−1 [−2, 1]	0 [−4, 5]	2 [0, 4]	2 [0, 5]	1 [−2, 4]
Adjusted R^2^ - full model	46 %	23 %	20 %	39 %	22 %	18 %	56 %
B)							
2nd trimester hormone only	57 [35, 83]	76 [45, 114]	57 [36, 82]	103 [72, 140]	44 [29, 60]	64 [36, 98]	61 [45, 78]
Adjusted R^2^ - basic model	42 %	40 %	45 %	63 %	55 %	36 %	71 %
2nd trimester hormone							
Age at blood draw	−1 [−3, 2]	0 [−4, 5]	0 [−2, 2]	1 [−4, 6]	−1 [−3, 1]	−3 [−7, 0]	−3 [−6, 1]
Smoking	27 [2, 57]	21 [−22, 79]	20 [0, 44]	59 [1, 151]	−26 [−50, −5]	−1 [−41, 37]	−21 [−83, 25]
Parity	−2 [−21, 16]	2 [−30, 37]	−17 [−36, 0]	−8 [−63, 40]	16 [1, 34]	12 [−12, 42]	−8 [−39, 19]
Child sex	8 [−9, 27]	−8 [−44, 24]	4 [−12, 22]	−10 [−52, 27]	2 [−15, 18]	7 [−18, 35]	−4 [−34, 23]
Birth length [per 5 cm]	−10 [−31, 9]	−20 [−63, 14]	−11 [−31, 6]	11 [−26, 57]	18 [1, 38]	14 [−14, 48]	24 [−5, 62]
Birth weight [per 100 g]	−1 [−3, 1]	−1 [−4, 2]	−1 [−2, 0]	0 [−3, 3]	1 [0, 2]	1 [−1, 4]	2 [−1, 4]
Adjusted R^2^ - full model	51 %	39 %	54 %	70 %	67 %	31 %	70 %
C)							
1st & 2nd trimester hormones							
Adjusted R^2^ - basic model	45 %	38 %	45 %	61 %	57 %	37 %	70 %
Adjusted R^2^ - full model	55 %	38 %	52 %	67 %	69 %	32 %	69 %

^a^ All models include 1st or 2nd trimester hormone concentration and the respective gestational age

Second trimester hormone concentrations explained 36 % (OPG) to 71 % (sRANKL) of the variation in the 3rd trimester on their own. Addition of maternal or child characteristics resulted in changes similar to those observed using 1st trimester concentrations (Table 4B), with slightly increased adjusted R^2^ statistics for testosterone, estradiol, progesterone, and PRL (7 % for testosterone to 12 % for PRL).

Models that included both 1st and 2nd trimester hormone concentrations but no other variables accounted for 37 % (OPG) to 70 % (sRANKL) of the variation observed in the 3rd trimester (Table 4C); results were similar after addition of all maternal and newborn characteristics.

Finally, we examined the extent to which 2nd and 3rd trimester hormone concentrations were associated with 1st trimester concentrations: second trimester hormone concentrations explained 25 % (estradiol and PRL) to 73 % (sRANKL) of the variation in the 1st trimester on their own, with slightly improvements after addition of maternal or child characteristics (Additional file 1A). Third trimester hormone concentrations explained <31 % (all hormones except sRANKL) of the variation in the 1st trimester on their own (Additional file 1B). Addition of 3rd trimester hormone concentrations to models based on 2nd trimester hormone concentrations did not improve adjusted R^2^ statistics (Additional file 1C).

### Two consecutive pregnancies

Next, we compared hormone concentrations during each trimester in women providing samples from two consecutive pregnancies. The correlations between two consecutive pregnancies for individual hormones by trimester are presented in Table 5. First trimester progesterone concentrations (*r* = 0.47; *p* < 0.05) were most strongly correlated between pregnancies, whereas for estradiol and testosterone the between-pregnancy correlations were strongest in the 3rd trimester (r_E2_ = 0.68, r_T_ = 0.69; *p* < 0.01). Concentrations of estrone were well correlated in all trimesters (*r* > 0.59; *p* < 0.01). Using the Bonferroni corrected p-value of 0.0003, 1st trimester estrone remained significantly correlated between pregnancies.

**Table 5:** Spearman correlation coefficients adjusted for gestational age in the trimesters between two successive pregnancies

Trimester	Estradiol	Estrone	Progesterone	Testosterone
1	0.467	0.761 ^*^	0.469 ^***^	0.495 ^***^
2	0.353	0.592 ^**^	−0.214	0.526 ^***^
3	0.683 ^**^	0.717 ^**^	0.371	0.694 ^**^

Significant *p*-values are indicated as ^*^ < 0.0003 (Bonferroni corrected *p*-value), ^**^ < 0.01, ^***^ < 0.05

Median changes between the two pregnancies were low (Table 6) and showed no significant differences with the exception of testosterone in the 3rd trimester (T_3 pregnancy 1_ = 1.04 ng/mL, T_3 pregnancy 2_ = 0.89 ng/mL; p_WS_ = 0.02). When comparing testosterone concentrations by parity, primiparous women had higher 3rd trimester testosterone levels than multiparous women (T_3 primi_ = 1.04 ng/mL, T_3 multi_ = 0.98 ng/mL; p_WS_ = 0.007). However, no further decrease in 3rd trimester testosterone levels was evident when comparing multiparous women to women with three children (T_3 multi_ = 0.98 ng/mL, T_3 3 ch_ = 0.94 ng/mL; p_ws_ = 0.31).

**Table 6:** Median hormone concentrations (10th-90th percentiles) and median change by trimester in 21 consecutive pregnancies ^a^

	Pregnancy 1	Pregnancy 2	Median change	p_WS_^b^
Estradiol (ng/mL)				
1st trimester	2.32 (1.27–3.76)	1.98 (1.11–3.58)	−0.3 (−1.3; −1.3)	0.10
2nd trimester	9.00 (5.91–13.3)	7.96 (5.21–13.2)	−0.3 (−0.9; 1.3)	0.18
3rd trimester	22.6 (16.2–34.2)	20.0 (14.2–32.0)	−0.2 (−0.9; 0.5)	0.07
Estrone (ng/mL)				
1st trimester	0.73 (0.42–1.60)	0.73 (0.28–1.50)	−0.2 (−1.1; 0.5)	0.11
2nd trimester	4.19 (2.03–6.76)	3.90 (1.67–8.17)	−0.2 (−1.0; 0.4)	0.10
3rd trimester	8.00 (4.34–17.1)	7.80 (3.32–12.2)	−0.3 (−0.6; 0.2)	0.08
Progesterone (ng/mL)				
1st trimester	30.6 (22.1–47.4)	31.2 (22.9–45.0)	0 (−0.5; 0.6)	0.65
2nd trimester	56.9 (39.3–85.3)	62.3 (40.4–78.7)	−0.1 (−0.8; 1.0)	0.96
3rd trimester	161 (127–207)	165 (123–225)	0.1 (−0.7; 0.5)	0.55
Testosterone (ng/mL)				
1st trimester	0.84 (0.52–1.38)	0.78 (0.46–1.43)	0 (−1.2; 0.5)	0.31
2nd trimester	1.10 (0.68–1.67)	1.02 (0.55–1.50)	−0.2 (−0.9; 0.6)	0.09
3rd trimester	1.04 (0.60–2.88)	0.89 (0.60–1.45)	−0.4 (−1.0; 0.2)	0.002

^a^ Conversion from ng/ml to nmol/l (SI units): estradiol*3.671, estrone*3.699, progesterone*3.18, testosterone*3.467, and prolactin*0.04348

^b^ *p*-value obtained from the Wilcoxon signed rank sum test

### Sensitivity analyses (data not shown)

Results did not change when limiting the study population to primi- and multi-gravid women.

The presented results for testosterone are based on all measurements. Excluding the testosterone measurements from the batch that resulted in higher inter-batch CVs for this hormone (*n* = 80 samples) did not change the direction or significance of the associations with the exception of attenuated results for 3rd trimester levels by parity (original: T_3 primi_ = 2.56 ng/mL vs. T_3 multi_ = 0.71 ng/mL, p_MW_ < 0.01; limited: T_3 primi_ = 2.16 ng/mL vs. T_3 multi_ = 0.69 ng/mL, p_MW_ = 0.13).

Sensitivity analyses were also conducted replacing missing sRANKL values (*n* = 21 samples) with the lower detection limit of 0.02 pmol/L. This approach did not change the direction or significance of the associations (e.g., original: T_1 primi_ = 0.32 pmol/L vs. T_1 multi_ = 0.56 pmol/L, p_MW_ = 0.04; replaced T_1 primi_ = 0.22 pmol/L vs. T_1 multi_ = 0.53 pmol/L, p_MW_ = 0.04).

## Discussion

To our knowledge, we provide the first observational data on correlations between hormone concentrations both across trimesters of a single pregnancy, as well as between two subsequent pregnancies. Our data show that third trimester hormone concentrations can be estimated from concentrations measured in the 1st or 2nd trimester, and that including maternal and fetal covariates, most notably parity, smoking, and fetal sex improved the estimates.

### Sex steroids

During the first 9 weeks of pregnancy the corpus luteum and, to a lesser extent, the maternal ovary and the adrenal cortex, contribute to circulating concentrations of maternal estradiol, estrone, and progesterone. After this period, the placenta becomes the predominant source of maternal steroids [16]. The substantial increase in maternal serum concentrations of estradiol, estrone, and progesterone throughout pregnancy is well established and was also evident in our study [16, 17]. The weaker and gradual increase in testosterone concentration across gestation has also been reported in prior longitudinal studies [17, 18].

Estradiol concentrations were correlated in two successive pregnancies in our investigation, with stronger coefficients in the 3rd (*r* = 0.68) as compared to the 1st (*r* = 0.47) trimester. A previous study based on 34 women with uncomplicated first and second full-term pregnancies reported a strong correlation (*r* = 0.78) between early estradiol levels in the consecutive pregnancies [19].

We observed a non-significant suggestion of lower estradiol concentrations throughout pregnancy in multiparous women. This is consistent with previous studies reporting lower first and second trimester estradiol levels in multiparous as compared to primiparous women [20–24]. The decreased concentrations of estradiol in multiparous women are hypothesized to be a result of increased pregnancy-induced 16α-hydroxylase activity in the maternal liver during earlier pregnancies leading to increased metabolism of estradiol and thus lower circulating estradiol in subsequent pregnancies [25]. We observed significantly lower progesterone and testosterone concentrations in multiparous as compared to primiparous women; this was also reported in prior studies on early pregnancy hormones [21–24].

Women who smoked during pregnancy had higher 3rd trimester estradiol levels than non-smoking women. Prior studies based on 1st or 2nd trimester measurements reported no association of estradiol concentrations with smoking [21, 22, 24]. In agreement with our results, higher testosterone levels in smoking women were also observed in one [22] of two studies [24].

We observed no differences in hormone concentrations between women pregnant with a boy or a girl. Prior studies are inconsistent, with some reporting an increase in early pregnancy estradiol and testosterone concentrations in women pregnant with a girl [22, 23], and others observing no differences by fetal gender [21, 26]. A decrease in pregnancy progesterone levels in women carrying a female fetus was observed in one study that collected blood at gestational weeks 16 and 27 [21], but not in studies conducted in gestational weeks 6–20 [22, 23, 27].

### Prolactin

Prolactin is produced by the pituitary gland in a pulsatile fashion and plays essential roles in mammary development and lactation, as well as reproduction [28]. Though the decidua or the fetus cannot be ruled out as a potential source of PRL during pregnancy, it is believed that the maternal pituitary is the main source of PRL secretion in pregnant women [29].

We observed an increase in PRL levels with progressing pregnancy; this is consistent with prior longitudinal studies [17, 30] and a cross-sectional study [29]. The increase in PRL concentration is believed to result from the stimulatory effect of estradiol, and to a lesser extent progesterone, on the pituitary gland [29].

Contrary to previous reports, we did not observe lower prolactin levels in multiparous as compared to primiparous women [20, 30]. Estrogens are hypothesized to stimulate the enlargement of the pituitary gland during pregnancy resulting in increased PRL concentrations, which parallel the increase in estrogens [29, 31]. In our study, the lack of an association between parity and PRL may be explained by the similar estradiol concentrations irrespective of parity. Two prior studies showing a decrease in PRL also observed decreased estradiol concentrations in multiparous women [20, 24].

Past studies reported lower early pregnancy PRL in women smoking during pregnancy as compared to non-smoking women [24, 30]. We observed a non-significant suggestion of lower PRL concentrations in smoking women in the 1st and 2nd trimester and significantly lower PRL levels in the 3rd trimester. Given that nicotine exposure induces dopamine release, which inhibits PRL secretion, smoking may cause reduced PRL levels [32].

We further observed significantly lower first trimester PRL levels in women pregnant with a boy as compared to women pregnant with a girl, whereas the lower levels in the 2nd or 3rd trimester were not significantly different. Prior studies investigating gender-related differences are inconclusive and reported lower levels in women carrying a girl (gestational weeks 16, 27, 34, or 36) [30, 33] or no association with fetal sex (gestational weeks 6–20, 28–32, 33–36, or 40) [34, 35]. A recent longitudinal study showed decreased PRL concentrations in women pregnant with a girl in gestation weeks 9–17 and increased concentrations at delivery [27]. A plausible explanation for our findings is difficult to formulate as no consistent pattern is evident from available data. It is likely that the observed changes are not gender-related but due to other factors influencing PRL levels, such as stress or fasting status. However, data on these factors were not available for our study.

### OPG and sRANKL

OPG and sRANKL are members of the tumor necrosis factor family and are involved in the regulation of bone resorption and bone mass [36].

The observed increase in OPG serum concentrations during pregnancy in our data has been reported in other longitudinal [37–39] and cross-sectional studies [40]. The main source of maternal serum OPG during pregnancy is unclear, however the placenta and breast tissue express high amounts of OPG [37–39]. Concentrations of OPG did not vary with neonatal gender or parity in our study, consistent with a prior report [40].

We did not see any change in sRANKL concentrations during pregnancy in our data. To our knowledge, the only other study on sRANKL in pregnancy observed lower concentrations in late pregnancy as compared to pre-conception levels (mean at week 36 = 0.77 vs. mean before pregnancy = 1.03; *p* = 0.03) [38]. We observed higher first trimester sRANKL concentrations in multiparous women than in primiparous women; to our knowledge this has not been previously reported.

Birth length, and to a lesser extent birth weight, were weakly correlated with OPG and sRANKL. To our knowledge, the only other study available to date observed positive correlations of child’s length and weight with sRANKL, but not with OPG concentrations at birth [41].

Our study has a number of strengths: 1) we specified 2-week ranges in each trimester for sample collection to account for the changing hormone concentrations during pregnancy; 2) with the equal number of primiparous and multiparous women, we were able to investigate hormone variations by parity; and 3) we focused on uncomplicated pregnancies with a healthy singleton offspring, as pregnancy-related complications (e.g. pre-eclampsia) can cause changes in the hormonal milieu.

One limitation is that sRANKL could not be quantified in 21 samples (14 %), as values were below the limit of detection. This might be because the majority of RANKL is cell bound and therefore not detectable in the circulation [42]. However, sRANKL concentrations did not change during pregnancy and results based on the original values were almost identical when compared to those replacing the missing measurements by 0.02 pmol/L; the lower limit of detection of the assay. Another limitation is that smoking status was assessed during the first trimester only. As more than 60 % of women who smoke in the first trimester are reported to continue throughout pregnancy, [43, 44] the observed changes in estradiol, testosterone, and PRL levels are thus likely to represent concentrations in current smokers. Furthermore, we cannot rule out that some of our findings are due to chance as the sample size in some of the subgroup analyses was limited. Finally, while results of this study are informative for research purposes, they are not of clinical significance.

## Conclusions

Our observational study shows that hormone concentrations measured in the 1st, 2nd, or 3rd trimester are correlated, as are hormone concentrations between pregnancies. Furthermore, to some extent it is possible to estimate selected 3rd trimester hormone concentrations based on one hormone measurement drawn in early gestation and pregnancy characteristics, whereas it appears to be less reliable to estimate early pregnancy concentrations using measurements from late pregnancy. As it is hypothesized that pregnancy is related to disease later in the mother and/or the offspring, the hormonal milieu of pregnancy is an area of interest for further investigation. Longitudinal studies investigating hormonal patterns from pre-conception through postpartum should be considered.

## Abbreviations

CV, coefficient of variation; OPG, osteoprotegerin; PRL, prolactin; sRANKL, soluble receptor activator of nuclear factor kappaB ligand

## Additional file

Additional file 1: Percentage changes [95 % confidence intervals] in 1st trimester hormone concentrations given 2nd and 3rd trimester concentrations and maternal and newborn characteristics. (DOCX 21 kb)
