# Supplementary material for: Hormone concentrations throughout uncomplicated pregnancies: a longitudinal study
Source: BMC Pregnancy Childbirth. 2016 Jul 4;16:146. doi: 10.1186/s12884-016-0937-5 (PMC4932669; doi:10.1186/s12884-016-0937-5)
Supplement: Additional file 1: — Percentage changes [95 % confidence intervals] in 1st trimester hormone concentrations given 2nd and 3rd trimester concentrations and maternal and newborn characteristics. (DOCX 21 kb) [file 12884_2016_937_MOESM1_ESM.docx]

**Additional file 1. Percentage changes [95% confidence intervals] in 1^st^ trimester hormone concentrations given 2^nd^ and 3^rd^ trimester concentrations and maternal and newborn characteristics ^a^**

|  | **Estradiol** | **Estrone** | **Progesterone** | | **Testosterone** | | **Prolactin** | **OPG** | **sRANKL** |
| --- | --- | --- | --- | --- | --- | --- | --- | --- | --- |
| **A)** |  |  |  | |  | |  |  |  |
| 2^nd^ trimester hormone only | 50 [24; 82] ^b^ | 52 [25; 84] ^a^ | 48 [24; 75] ^a^ | | 63 [42; 86] ^a^ | | 54 [25; 90] ^b^ | 86 [55; 124] ^a^ | 85 [64; 108] ^a^ |
| *Adjusted R^2^ - basic model* | *25%* | *29%* | *28%* | | *55%* | | *25%* | *47%* | *73%* |
| 2^nd^ trimester hormone |  |  |  | |  | |  |  |  |
| Age at blood draw | 3 [1; 6] ^b^ | 2 [-2; 6] | 1 [-1; 4] | | 2 [-1; 6] | | -2 [-6; 2] | -2 [-6; 1] | 4 [-0; 8] ^c^ |
| Smoking | -3 [-36; 28] | -30 [-86; 10] | -4 [-33; 22] | | -5 [-55; 40] | | 4 [-44; 55] | 25 [-10; 72] | -14 [-84; 41] |
| Parity | 1 [-22; 24] | 19 [-11; 57] | -13 [-37; 6] | | -28 [-78; 8] | | -43 [-87; -9] ^b^ | -11 [-41; 13] | 25 [-6; 65] |
| Child sex | -20 [-47; 2] ^c^ | -33 [-73; -1] ^b^ | -3 [-24; 17] | | -4 [-36; 25] | | -37 [-82; -4] ^b^ | -13 [-43; 12] | -15 [-52; 15] |
| Birth length [per 5 cm] | 7 [-17; 34] | 26 [-7; 71] | -4 [-27; 18] | | -3 [-36; 28] | | 8 [-26; 48] | -19 [-54; 9] | 20 [-14; 63] |
| Birth weight [per 100 g] | 0 [-2; 2] | 2 [-0; 5] ^c^ | -1 [-2; 1] | | -1 [-3; 2] | | 2 [-1; 5] | -2 [-4; 1] | 1 [-2; 4] |
| *Adjusted R^2^ - full model* | *35%* | *44%* | *28%* | | *53%* | | *44%* | *47%* | *76%* |
|  |  |  |  | |  | |  |  |  |
| **B)** |  |  |  | |  | |  |  |  |
| 3^rd^ trimester hormone only | 23 [-2; 54] ^c^ | 25 [6; 49] ^b^ | | 30 [8; 57] ^b^ | 35 [19; 54] ^a^ | 48 [7; 104] ^b^ | | 45 [20; 76] ^b^ | 94 [63; 132] ^a^ |
| *Adjusted R^2^ - basic model* | *4%* | *12%* | *11%* | | *31%* | | *8%* | *23%* | *58%* |
| 3^rd^ trimester hormone |  |  | |  |  |  | |  |  |
| Age at blood draw | 3 [-1; 6] | -0 [-5; 5] | | 1 [-2; 4] | -0 [-5; 4] | 0 [-5; 5] | | -0 [-4; 4] | 5 [-0; 10] ^c^ |
| Smoking | -4 [-46; 35] | -33 [-100; 13] | | -15 [-45; 10] | 3 [-59; 67] | 19 [-38; 95] | | 25 [-16; 82] | -24 [-124; 45] |
| Parity | -2 [-31; 25] | 15 [-20; 59] | | -11 [-38; 11] | -59 [-125; -12] ^b^ | -69 [-132; -23] ^b^ | | -3 [-37; 30] | 47 [5; 106] ^b^ |
| Child sex | -18 [-50; 7] | -29 [-75; 6] | | -3 [-25; 18] | -3 [-42; 35] | -51 [-106; -11] ^b^ | | -7 [-42; 23] | -11 [-58; 29] |
| Birth length [per 5 cm] | 8 [-20; 40] | 31 [-8; 84] | | 7 [-15; 32] | -7 [-51; 32] | 5 [-39; 54] | | -7 [-46; 28] | 13 [-33; 71] |
| Birth weight [per 100 g] | 1 [-1; 3] | 3 [-0; 6] ^c^ | | 0 [-1; 2] | -1 [-4; 3] | 2 [-2; 5] | | -1 [-4; 2] | 1 [-3; 5] |
| *Adjusted R^2^ - full model* | *18%* | *28%* | *19%* | | *33%* | | *37%* | *18%* | *59%* |
|  |  |  | |  |  |  | |  |  |
| **C)** |  |  | |  |  |  | |  |  |
| 2^nd^ & 3^rd^ trimester hormones |  |  | |  |  |  | |  |  |
| *Adjusted R^2^ - basic model* | *23%* | *25%* | *23%* | | *53%* | | *24%* | *47%* | *78%* |
| *Adjusted R^2^ - full model* | *33%* | *44%* | | *30%* | *50%* | *45%* | | *46%* | *78%* |

^a^ All models include 2^nd^ or 3^rd^ trimester hormone concentration and the respective gestational age.
